# Understanding sexual health service access for gay, bisexual and other men who have sex with men in Ireland during the COVID-19 crisis: Findings from the EMERGE survey

**DOI:** 10.1371/journal.pone.0306280

**Published:** 2024-07-01

**Authors:** Adam Shanley, Kate O’Donnell, Peter Weatherburn, John Gilmore, T. Charles Witzel

**Affiliations:** 1 HIV Ireland, Dublin, Ireland; 2 HSE Health Protection Surveillance Centre, Dublin, Ireland; 3 London School of Hygiene and Tropical Medicine, London, United Kingdom; 4 University College Dublin, Dublin, Ireland; 5 University College London, London, United Kingdom; University of Technology Sydney, AUSTRALIA

## Abstract

**Background:**

In the Republic of Ireland, the COVID-19 crisis led to sexual health service closures while clinical staff were redeployed to the pandemic response. Gay, bisexual and other men who have sex with men (gbMSM) face pre-existing sexual health inequalities which may have been exacerbated. The aim of this study is to understand sexual health service accessibility for gbMSM in Ireland during the COVID-19 crisis.

**Methods:**

EMERGE recruited 980 gbMSM in Ireland (June-July 2021) to an anonymous online survey investigating well-being and service access through geo-location sexual networking apps (Grindr/Growlr), social media (Facebook/Instagram/Twitter) and collaborators. We fit multiple regression models reporting odds ratios (ORs) to understand how demographic and behavioural characteristics (age, sexual orientation, HIV testing history/status, region of residence, region of birth and education) were associated with ability to access services.

**Results:**

Of the respondents, 410 gbMSM accessed sexual health services with some or no difficulty and 176 attempted but were unable to access services during the COVID-19 crisis. A further 382 gbMSM did not attempt to access services and were excluded from this sample and analysis.

Baseline: mean age 35.4 years, 88% gay, 83% previously tested for HIV, 69% Dublin-based, 71% born in Ireland and 74% with high level of education.

In multiple regression, gbMSM aged 56+ years (aOR = 0.38, 95%CI:0.16, 0.88), not previously tested for HIV (aOR = 0.46, 95%CI:0.23, 0.93) and with medium and low education (aOR = 0.55 95%CI:0.35, 0.85) had lowest odds of successfully accessing services.

GbMSM with HIV were most likely to be able to access services successfully (aOR = 2.68 95%CI:1.83, 6.08).

Most disrupted services were: STI testing, HIV testing and PrEP.

**Conclusions:**

Service access difficulties were found to largely map onto pre-existing sexual health inequalities for gbMSM. Future service development efforts should prioritise (re)engaging older gbMSM, those who have not previously tested for HIV and those without high levels of education.

## Introduction

The COVID-19 pandemic caused enormous disruption to nearly every aspect of daily life in countries across the globe [1–6]. Governments of many countries implemented strict controls over citizens in an early effort to minimise the impact on their social, economic and healthcare systems [7,8].

The first case of COVID-19 disease was identified in Ireland in late February 2020. By March 2020, the Irish government had closed schools, cultural institutions and cancelled large public gatherings [9,10]. Following, emergency legislation was passed, giving the state powers to implement lockdowns and other restrictions aimed at curbing community transmission of the virus and minimising pressure on health service resources.

Strict government lockdowns were implemented throughout 2020 and 2021 with the first lasting 3 months. These measures, aimed at reducing social contact and slowing the spread of the virus, involved the closure of businesses deemed non-essential, a ban on movement beyond a 2-kilometre radius of home, limited gatherings outdoors with no indoor congregating, and the implementation of remote work where possible. Travel restrictions were also enforced, with strict guidelines in place for those entering and leaving the country. Additionally, mask mandates were widely implemented in the remaining indoor public spaces, and individuals were advised to maintain physical distancing and practice good hygiene at all times [10]. COVID-19 cases and related mortality dropped to low levels by mid-June 2020 [10], resulting in an easing of lockdown and introduction of restrictions such as mandatory mask-wearing in public places. Ireland re-entered lockdown at the end of December 2020, days before the nation’s vaccination programme commenced [10,11]. Restrictions remained in place until May 2021 with cases rising again in July. Restrictions on hospitality and gatherings continued with proof of vaccination a mandatory requirement to enter indoor hospitality and for travel [11]. Further easing of restrictions in December 2021 resulted in significant spread of the Omicron variant. A final national lockdown was implemented from 26^th^ of December 2021 until easing of almost all COVID-19 restrictions from 22^nd^ of January 2022 [11].

While the lives of all people living in Ireland were impacted by the COVID-19 pandemic and government restrictions, minority populations likely experienced more pronounced negative impacts [12]. Gay, bisexual and other men who have sex with men (gbMSM) are one such minority and are disproportionately affected by HIV and other sexually transmitted infections (STIs) [13,14]. In the decade leading up to the COVID-19 pandemic numerous studies have highlighted the need to respond to this disparity with investment and expansion of available sexual health services and implementation of new models of sexual health care, including community-based testing and wider provision of HIV pre-exposure prophylaxis (PrEP) [14–17]. While progress had been made in achieving this, pandemic-related restrictions have likely worsened sexual health outcomes, driven by disruption to service access.

Evidence suggests limits on social mixing have had detrimental impacts on mental health in Ireland [5,18]; gbMSM may be more vulnerable as this population often has weaker social ties and face greater levels of financial precarity, something which may be especially true for certain subgroups such as older men, those living in rural areas, those with low and medium levels of education and migrants [19–23]. This may have downstream impacts HIV and STI risk, ability to access services, as well as mental well-being and substance use. GbMSM from Latin American backgrounds are a critical priority group in Ireland in the context of vulnerability to issues around poor sexual health and well-being [17,24]. The COVID-19 crisis may have had greater impacts on this group when compared to White Irish gbMSM given their pre-existing vulnerabilities related to employment, weaker social ties, and marginalisation all of which can shape sexual health and well-being. New and escalating levels of substance use during the pandemic has been observed in many settings [25–27]. It is plausible that this will disproportionately impact gbMSM in Ireland given the higher rates of substance use observed in this group compared to the general population prior to COVID-19 [24,28].

Sexual health care was highlighted by the World Health Organization as an essential service to maintain during the COVID-19 pandemic [29]. However, many clinics faced substantial disruption with significant reductions and sometimes complete closures [12]. This was driven in large part by the redeployment of infectious disease and genitourinary medicine specialists and sexual health service staff to assist in the COVID-19 response. This likely led to a decrease in testing opportunities and potentially longer waiting times for STI treatment [12]. Prevention interventions (e.g. PrEP and HIV post-exposure prophylaxis (PEP)) may have been interrupted due to sexual health service access issues. Such disruption to testing, treatment and prevention behaviours adds to the potential for onward transmission of STIs and a further deepening of sexual health disparities for and among gbMSM.

Understanding the potential effects of the COVID-19 crisis on sub-groups of gbMSM is essential for planning sexual health services in the near and medium terms. Recovery efforts must address inequalities that existed previously and those exacerbated by the pandemic. The aim of this research is therefore to understand sexual health service accessibility for gbMSM in Ireland during the COVID-19 crisis. We do this by identifying demographic and behavioural characteristics associated with attempting and being unable to access services during the COVID-19 crisis in Ireland and by describing which services were most disrupted.

## Materials and methods

### Eligibility and recruitment

The EMERGE (**e**ffect of COVID-19 and govern**me**nt **r**estriction on the sexual health and well-being of **g**ay and bis**e**xual men) survey was conducted between June 5^th^ and July 4^th^ 2021, when social distancing restrictions were substantially lifted and hospitality venues were re-opening and prior to the final national lockdown. EMERGE recruited men (cis and trans), aged 18+, who lived in Ireland for some or all of the COVID-19 pandemic, and who reported sexual attraction to other men (cis and/or trans) or lifetime sex with other men (cis and/or trans). Recruitment was online through social media (Facebook, Instagram and Twitter), geo-location social/sexual networking applications (apps) (Grindr, Scruff and Growlr), mailing lists of organisations and the LGBTQ press.

We conducted patient and public involvement by inviting 10 gbMSM (n = 6 English, n = 2 Portuguese and n = 2 Spanish speaking) to assess comprehension and test survey logic. On completion of the final report (see Witzel et al 2022 [12]), we developed a community report which was launched at an event held at an LGBT+ social venue. A social media campaign followed to relay the findings to the wider community.

### Survey design and measures

EMERGE was available in English, Spanish and Portuguese. Survey items were drawn from prior cross-sectional studies for ease of comparison [24,30], with COVID-19 specific questions developed by the researchers.

Demographic and behavioural characteristics treated as independent variables include age, sexual orientation, HIV status (positive, negative or untested), region of residence, region of birth and highest educational qualification.

Age was recoded into groups (18–25; 26–35; 36–45; 46–55; 55+). Sexual orientation was recorded as gay, bisexual, other and straight; other and straight were combined for analysis. Highest educational attainment was recoded into medium and low (no educational qualification, Intermediate, Junior or Group Certificate, Leaving Certificate, Higher education below degree level) and high (degree or higher). For region of residence, responses were reorganised from the 26 counties of Ireland to Dublin, rest of Leinster, Munster, Connaught and Ulster. To explore the specific health needs of gbMSM from Latin America and the Caribbean (a priority group in the Irish HIV response), country of birth was recoded from individual country to Ireland, Latin America and the Caribbean or rest of world.

To measure health service access, respondents without diagnosed HIV (those who had most recently tested negative or who had not had an HIV test) were asked: “During the COVID-19 Crisis have you been able to access sexual health services (including access to PrEP, HIV/STI testing, PEP, condoms and lube and advice/information/support)”. Respondents who reported having diagnosed HIV were asked the same question but with a modified list of examples (STI testing, condoms and lube and advice/information/support). Response options for both included:

*Yes*, *I have accessed sexual health services with no difficulty*; *Yes*, *I have accessed sexual health services but not as easily as usual; Yes*, *I have accessed sexual health services*, *but not the one I wanted; No*, *I have tried and wasn’t able to access sexual health services; No*, *I have not tried to access sexual health services*.

Responses to both questions were combined, and the variable recoded to ‘Yes, with or without difficulty’; ‘No, was unable to access sexual health services’ and ‘Did not attempt.’ Those who did not attempt to access services were excluded from our analysis.

Those who reported difficulty with sexual health service access were asked ‘Which services were you attempting to access’; with different options for HIV negative/untested and gbMSM living with HIV. These were combined into one variable.

### Analysis

After excluding gbMSM who did not attempt to access services, we tabulated baseline characteristics of the remaining sample.

We used logistic regression to understand how demographic and behavioural variables were associated with our dependent variable of ability to access sexual health services during the COVID-19 pandemic. We regressed our dependent variable on age, sexual orientation, HIV status, region of residence, country of birth and highest educational qualification. We chose reference categories to elucidate key health equity dimensions in gbMSM in Ireland (e.g. high educational attainment, HIV negative status, having been born in Ireland) [24,30]. We included each independent variable and our dependent variable into a bivariate logistic regression model using block entry. In order to focus on elucidating the impact of COVID-19 on existing health inequalities in Ireland, we did not include a sexual risk or sexual behaviour variable as all gbMSM in our analytic sample experienced service access need. We report odds ratios for all models.

Finally, we tabulated which services respondents had the most difficulty accessing.

Missing data across all included variables was <5%. We assessed this level of missing data to be acceptable for a cross-sectional community-based survey and did not attempt corrective statistical procedures [31]. Data were analysed in Stata V17.

## Results

### Baseline

EMERGE recruited 984 gbMSM, 4 of whom were later excluded as found to be ineligible. Excluding 382 gbMSM who did not attempt to access services and 12 who did not respond to the relevant service access questions, the final analytic sample for our study was 586.

Overall, 176 (30.0%) gbMSM attempted but were unable to access sexual health services during the COVID-19 crisis and 410 (70.0%) accessed services with some or no difficulty. Table 1 presents our analytic sample. Table 1 provides the full EMERGE sample.

**Table 1:** Description of analytic sample, EMERGE survey 2021.

Characteristic	All n	Could not access services n(%)	Accessed services n(%)
All	586	176 (100.0)	410 (100.0)
**Age**			
18–25	70	27 (38.6)	43 (61.4)
26–35	266	65 (24.4)	201 (75.6)
36–45	144	43 (29.9)	101 (70.1)
46–55	72	26 (36.1)	46 (63.9)
56+	34	15 (44.1)	19 (55.9)
**Sexual Orientation**			
Gay	518	156 (30.1)	362 (69.9)
Bisexual	60	19 (31.7)	41 (68.3)
Other/straight	8	1 (12.5)	7 (87.5)
**HIV status**			
Negative	487	147 (30.2)	340 (69.8)
Positive	57	8 (14.0)	49 (86.0)
Untested	42	21 (50.0)	21 (50.0)
**Region**			
Dublin	397	116 (29.2)	281 (70.8)
Rest of Leinster	63	18 (28.6)	45 (71.4)
Munster	78	18 (23.1)	60 (76.9)
Connaught & Ulster	37	18 (48.6)	19 (51.4)
**Country of birth**			
Ireland	412	131 (31.8)	281 (68.2)
LA & Caribbean	78	18 (23.1)	60 (76.9)
Other	87	22 (25.3)	65 (74.7)
**HEQ**			
High	426	112 (26.3)	314 (73.7)
Medium & low	148	60 (40.5)	88 (59.5)

### Regression analysis

In univariable logistic regression, those with least odds of successfully accessing services were gbMSM aged 18–25 years, 46–55 years, 56+ years, those who had not previously tested for HIV and those with medium and low levels of education. GbMSM with diagnosed HIV had the greatest odds of being able to access services during this period.

In multiple logistic regression, the odds of being able to access services were significantly lower in gbMSM aged 56+ years (versus those aged 25–34 years) (aOR = 0.38, 95%CI:0.16, 0.88), in those not previously tested for HIV (versus tested negative) (aOR = 0.46, 95%CI:0.23, 0.93) and in those with medium and low levels of education (versus those with higher level) (aOR = 0.55 95%CI:0.35, 0.85). GbMSM with diagnosed HIV remained the group with highest odds of ability to access services (versus those previously tested negative)(aOR = 2.68 95%CI:1.83, 6.08). This is presented below in Table 2.

**Table 2:** Service access among those who could not access services, EMERGE 2021.

Characteristic	Could not access services (n,%)	Unadjusted OR (95%CI)	Adjusted OR (95%CI)
**Age**			
18–25	27 (38.6)	0.52 (0.30; 0.90)*	0.74 (0.39; 1.42)
26–35	65 (24.4)	Ref	Ref
36–45	43 (29.9)	0.76 (0.48; 1.20)	0.77 (0.48; 1.26)
46–55	26 (36.1)	0.57 (0.33; 1.00)*	0.64 (0.35; 1.19)
56+	15 (44.1)	0.41 (0.20; 0.85)*	0.38 (0.16; 0.88)*
Chi^2^, df, p value		10.66, 4, 0.031	
**Sexual Orientation**			
Gay	156 (30.1)	Ref	Ref
Bisexual	19 (31.7)	0.93 (0.52; 1.70)	1.03 (0.54; 1.95)
Other / straight	1 (12.5)	3.02 (0.39; 24.72)	2.25 (0.26; 19.17)
Chi^2^, df, p value		1.45, 2, 0.48	
**HIV status**			
Negative	147 (30.2)	Ref	Ref
Positive	8 (14.0)	2.65 (1.22; 5.73)*	2.68 (1.83; 6.08)*
Untested	21 (50.0)	0.43 (0.23; 0.82)*	0.46 (0.23; 0.93)*
Chi^2^, df, p value		15.31, 2, <0.001	
**Region**			
Dublin	116 (29.2)	Ref	Ref
Rest of Leinster	18 (28.6)	1.03 (0.57; 1.86)	1.44 (0.75; 2.78)
Munster	18 (23.1)	1.38 (0.78; 2.43)	1.73 (0.93; 3.20)
Connaught & Ulster	18 (48.6)	0.44 (0.22; 0.86)*	0.57 (0.27; 1.17)
Chi^2^, df, p value		7.63, 3, 0.054	
**Country of birth**			
Ireland	131 (31.8)	Ref	Ref
LA & Caribbean	18 (23.1)	1.55 (0.88; 2.74)	1.28 (0.70; 2.36)
Other	22 (25.3)	1.38 (0.81; 2.33)	1.36 (0.77; 2.39)
Chi^2^, df, p value		577, 2, 0.182	
**HEQ**			
High	112 (26.3)	Ref	Ref
Medium & low	60 (40.5)	0.52.3 (0.35; 0.77)*	0.55 (0.35; 0.85)*
Chi^2^, df, p value		10.27, 1, 0.001	42.1, 14, <0.001

* P≤0.05.

### Service disruption

For those who were unable to access services, the services which they were attempting to access are outlined in Table 3. For gbMSM without diagnosed HIV, the most commonly disrupted services were: STI testing (75.6% n = 127), HIV testing (63.1% n = 106) and PrEP (52.1% n = 88). 6.5% (n = 11) reported difficulty accessing PEP. For gbMSM with diagnosed HIV, the most commonly disrupted service was STI testing (87.5%, n = 7).

**Table 3:** Most disrupted services, EMERGE 2021.

	STI testing	HIV testing	PrEP	PEP	Condoms and lube	Chemsex	Information & advice	Vaccinations	Other
HIV negative & unknown N = 168	127 (75.6)	106 (63.1)	88 (52.1)	11 (6.5)	27 (16.1)	2 (1.2)	19 (11.3)	29 (17.3)	1 (0.6)
Living with HIV N = 8	7 (87.5)	NA	NA	NA	3 (37.5)	0 (0)	3 (37.5)	3 (37.5)	1 (12.5)

## Discussion

This is the only study in Ireland exploring sexual health service access for gbMSM in Ireland during the COVID-19 pandemic. Sexual health services were already under strain when COVID-19 brought significant additional challenges. Undertaking our study at this time was an important opportunity to focus attention on the impact that reduced access to services can have in the response to the syndemic of STIs in Ireland that disproportionately affected gbMSM while responding to a new health threat [24,30,32]. In addition, our recruitment strategy engaged more gbMSM from Latin America and the Caribbean, a priority group in the HIV response in Ireland, than previous cross-sectional studies [24,30]. Understanding the scale of access issues and which sub-groups of gbMSM are most affected is critical in informing policy and designing responses which will aid recovery efforts targeting these groups.

We found that the COVID-19 pandemic and related government restrictions had a significant impact on access to and utilisation of sexual health services for gbMSM living in Ireland. Those with the least odds of successfully accessing services were gbMSM aged 56+ years, not previously tested for HIV and with medium and low levels of education. Conversely, gbMSM with diagnosed HIV were identified as the group with highest odds of successful access to sexual health services.

These results map broadly onto pre-existing health inequalities among gbMSM in Ireland and suggest that the difficulties in accessing services faced by gbMSM who are older, not previously tested and with lower levels of education likely were exacerbated during this period. Reasons are unclear but it is plausible that these groups had fewer cultural resources to draw upon in seeking sexual health care. Conversely, gbMSM with diagnosed HIV were most likely to be able to sustain sexual healthcare seeking, largely because HIV clinics remained open.

All gbMSM reporting difficulty with service access were asked what services they were attempting to access. Responses showed there was a very considerable impact on access to STI and HIV testing. While clinic-based services experienced the greatest degree of disruption in Ireland, the introduction of an HIV self-testing service by a community-based organisation in 2020 and the separate launch of an HIV & STI self-sampling service by the HSE in 2021, may have bridged the gap for some gbMSM (especially those who are most digitally literate) [33]. It is possible that a significant number of HIV and STI infections among gbMSM in Ireland may have gone undiagnosed as a result of diminished service access. Individuals who have missed opportunities to be linked to clinical care for treatment are at risk of experiencing poorer health outcomes. These missed opportunities increase the possibility of onward transmission with the potential to further widen health disparities.

Difficulty in PrEP access in Ireland pre-dates COVID-19. The national PrEP Programme was launched in November 2019, shortly before the pandemic and associated disruption. Strong demand for PrEP among gbMSM led to many services reaching capacity faster than expansion of services was possible. Many services considered alternative PrEP delivery pathways (e.g. remote consultations) in an effort to maintain access for those already in the programme [34]. However, our findings show that more than half of men without diagnosed HIV who experience service access difficulty (52.1%) were attempting to access PrEP. While unmet need was demonstrable before the pandemic, it has endured throughout the COVID-19 crisis and will require attention as a priority area as sexual health services stabilise.

Our findings show gbMSM living with HIV had the greatest odds of being able to access services. Maintenance of antiretroviral treatment for gbMSM living with HIV is critically important at both the individual and community level [35,36]. Avoiding disruption to viral suppression for the individual during this period will have maintained control over HIV-related morbidity and potential drug resistance, while at community level will have lessened the occurrence of HIV transmission, an outcome which would have had a disproportionate effect on vulnerable populations.

### Comparison with other research

Our study adds to a growing body of evidence of the disproportionate impact of the COVID-19 pandemic on sexual health service access for vulnerable populations globally, including gbMSM.

A study conducted in 30 countries across Africa, Asia, the Americas and Europe found COVID-19 restrictions hindered access to HIV and STI testing for 32.3% of people who needed it, highlighting sexual health service access during this period as a global issue [37].

Research from the UK found sexual health services were complex to access during the early stages of the pandemic, in line with our findings [38]. Further research conducted in 2021 found no increased testing need in the UK compared to 2017 following the lifting of most social restrictions [39]. The authors highlight the role of remote testing services, which were limited in Ireland at the time, underlining the role of service diversification in responding to shocks. Another UK study found that sub-groups of men and gender diverse people who have sex with men maintained or increased sexual behaviour, and (in contrast with our results) that younger people were more likely to have sexual health need during the early stages of the COVID-19 crisis [6,40].

Further, a survey in France conducted with gbMSM engaged in chemsex found that 25.3% had unmet sexual health testing need, and that this need was more pronounced in Asian men, indicating the need for country specific research highlighting new and accelerating health inequalities [41]. Finally, research from Germany highlights the potential impact of COVID-19 related sexual health service disruption, describing an increase in late diagnosis of HIV during social distancing restrictions [42].

### Recommendations

The data from this study provides important insights into the extent of unmet sexual health need during the COVID-19 pandemic. The following actions are recommended to address deficits in sexual health service accessibility and utilisation arising from pandemic restrictions.

#### a. Restore and improve sexual health services across Ireland and resource the expansion of their capacity to respond to a surge in testing and treatment needs

This study shows that the widespread disruption to sexual health services throughout Ireland has resulted in significant barriers to accessing sexual health services, including HIV and STI testing and–as evidenced by the national syphilis outbreak declared in 2021 and subsequent national surveillance data–has substantially impacted the number of STIs in the community [43]. Sexual health services before the COVID-19 pandemic were under resourced and accessibility was already suboptimal nationally. Capacity issues predate COVID-19 but were exacerbated by the pandemic and related government restrictions. These have likely also been worsened by the subsequent global mpox outbreak occurring primary among gbMSM and leading to substantial service difficulties in many settings [44–46]. Services must be appropriately resourced so that they can operate with sufficient capacity to respond to surges in testing demand and treatment needs while also expanding to cater for the substantial unmet demand which existed prior to the COVID-19 crisis.

#### b. Expand low threshold testing initiatives and invest in additional innovative HIV and STI testing strategies

Results from this study demonstrate the diversity of experience during the COVID-19 crisis of a variety of gbMSM who did not or were unable to access services. These results support the need for increasing emphasis on innovative strategies for HIV and STI testing to ensure it is as easy as possible for gbMSM to access testing. Some community-based rapid HIV testing services quickly reopened following the initial lockdown, and pilot initiatives (HSE Home STI Testing, MPOWER HIV Self-Test) were deployed and helped minimise service interruptions for those at risk of HIV and STI acquisition during subsequent waves of COVID-19 [33]. However, their geographical reach and capacity were limited. These services should be appropriately funded and expanded to be accessible throughout the country. Investment in additional innovative HIV and STI testing strategies should also be considered, particularly for those who prefer low threshold testing options and those who have not tested before.

#### c. Provide additional capacity to ensure equitable access to PrEP services across Ireland

Demand for PrEP in Ireland has been consistently high since the introduction of a National PrEP Programme in 2019, however, capacity to deliver this service to the many gbMSM who want and would benefit from it has been suboptimal. This research shows that more than half (52%) of the HIV negative men who had difficulty accessing services during restrictions were attempting to access PrEP, leading to significant missed HIV prevention opportunities. Access to PrEP is critically important, particularly given the continued increase in STIs. Enthusiasm in the uptake of PrEP potentially risks being diminished due to the sustained difficulty in accessing it. Without expansion of PrEP services, efforts to reduce new diagnoses in Ireland are likely unfeasible, as is the case in the UK [47]. Provision of additional capacity to ensure equitable access to PrEP services across Ireland is an urgent requirement.

#### d. Prioritise responses for those experiencing increased health inequalities

While health inequalities among and between groups of gbMSM existed before the pandemic, the results of this study suggest that inequalities may have intensified. Men living in rural areas, men with medium and low educational attainment, bisexual men and those who have experienced employment insecurity due to government restrictions are likely to be have experienced increasing health inequalities. All interventions targeting gbMSM should be especially mindful of their potential acceptability and accessibility to these groups. Prioritising responses addressing inequalities due to additional marginalisation must be a priority for sexual health programming going forward.

This study highlights the need to invest in responses that address deficits in sexual health service accessibility and utilisation arising from pandemic restrictions. Expanded efforts to identify at-risk individuals and link them to HIV & STI testing, treatment and prevention services are needed. Innovative approaches to sexual health service delivery must remain a key area of development. Responses that are devised should be designed with acceptability and accessibility for those experiencing increased health inequalities in mind. Additionally, consideration should be given to an “always-on” approach with the ability to flex and expand should the health service experience a significant reduction in the provision of services or shortfalls in capacity or staff due to a similar outbreak or system strain.

### Strengths and limitations

This is the only research in Ireland to have explored sexual health service access for gbMSM in Ireland during the COVID-19 pandemic. As such it provides vital evidence for policy makers, academics and commissioners. In addition, our recruitment strategy engaged more gbMSM from Latin America and the Caribbean (a priority group in the Irish HIV response) than previous cross-sectional studies. We note some limitations.

All outcomes are self-reported and therefore reflect participants’ understandings of their own experiences.

Recruitment made use of a range of online platforms in an attempt to capture diverse groups of gbMSM. Although gbMSM in Ireland are a highly digitally literate population with widespread internet use, individuals who abstain from social media or geo-location hook-up apps due to personal preferences, concerns about privacy, or discomfort with the platform, amongst other reasons, may not have been captured.

Data collection took place in June and early July of 2021. While the COVID-19 pandemic in Ireland dramatically changed following successful vaccination programmes in the summer of 2021, it has continued to change, shape and cause varying periods of disruption. Our results should therefore be understood as a snapshot of a particular period of time when disruption was beginning to reduce and health service provision was beginning to normalise.

For our sexual orientation category numbers describing their orientation as bisexual, other and straight are relatively small. We maintained a separate category of bisexual as these men are a key group in the Irish HIV response.

Our recruitment strategy reached very few men with low educational attainment (n = 31, 3.2%). Of these, 22 (71.0%) did not seek testing during the COVID-19 crisis and were excluded from our analytic sample. Because the remaining number in the sample was very small (n = 9) we combined men with low and medium levels of education. However, we acknowledge that gbMSM with low levels of education have both overlapping and distinct needs when compared to those with medium educational attainment. The specific experiences of this gbMSM with lower educational qualifications require concerted attention in future research.

## Conclusions

Our analysis demonstrates how the COVID-19 crisis and related government restrictions may have accelerated health inequalities in Ireland. We found that groups most likely to be unable to access sexual health services during the earlier stages of the pandemic were those ages 56+, who had not previously tested for HIV, and with medium and low levels of education. Conversely gbMSM with diagnosed HIV were most likely to be able to access services. As sexual health services continue to normalise, it is critical that future service development initiatives seek to (re)engage gbMSM who are older, not previously tested for HIV and without high levels of education.

## Supporting information

S1 Dataset (XLS)
